# Impact of Bevacizumab Relative Dose Intensity and Treatment-Induced Proteinuria on the Efficacy of Trifluridine/Tipiracil Plus Bevacizumab in Metastatic Colorectal Cancer

**DOI:** 10.3390/cancers18152429

**Published:** 2026-07-28

**Authors:** Tomoya Tsukida, Masatsune Shibutani, Shinya Nakatani, Hideki Tanda, Yuki Seki, Hiroaki Kasashima, Masanori Emoto, Kiyoshi Maeda

**Affiliations:** 1 Department of Gastroenterological Surgery, Graduate School of Medicine, Osaka Metropolitan University, Osaka 545-8585, Japan; sx25194n@st.omu.ac.jp (T.T.);; 2 Department of Nephrology, Graduate School of Medicine, Osaka Metropolitan University, Osaka 545-8585, Japan; 3 Department of Metabolism, Endocrinology and Molecular Medicine, Graduate School of Medicine, Osaka Metropolitan University, Osaka 545-8585, Japan

**Keywords:** metastatic colorectal cancer, bevacizumab, trifluridine/tipiracil, relative dose intensity, proteinuria, overall survival, adverse events

## Abstract

**Background**: Trifluridine/tipiracil (FTD/TPI) combined with bevacizumab (Bev) is an established later-line therapy for metastatic colorectal cancer (mCRC). However, the clinical impact of insufficient Bev dose intensity, often caused by cumulative renal toxicity, on treatment efficacy remains unclear. **Methods**: This retrospective cohort study evaluated 101 patients with mCRC receiving FTD/TPI + Bev to determine the clinical impact of Bev relative dose intensity (RDI). Based on a receiver-operating-characteristic analysis, a Bev RDI cutoff of 70% was established, categorizing patients into high-RDI (≥70%, n = 75) and low-RDI (<70%, *n* = 26) groups. **Results**: The high-RDI group demonstrated significantly longer progression-free survival (131 vs. 82 days, *p* = 0.003) compared to the low-RDI group. For overall survival, although statistical significance was not reached, a favorable trend was observed in the high-RDI group (343 vs. 215 days, *p* = 0.093). The disease control rate was also significantly higher in the high-RDI group (46.7% vs. 23.1%, *p* = 0.040). Treatment interruption due to proteinuria was the primary cause of reduced RDI. Furthermore, a history of severe proteinuria during prior anti-VEGF therapy strongly predicted its recurrence during the current regimen. **Conclusions**: Maintaining the dose intensity of Bev is crucial for maximizing the efficacy of FTD/TPI + Bev, highlighting the necessity of long-term proteinuria management across treatment lines.

## Simple Summary

A combination of drugs, trifluridine/tipiracil and bevacizumab, is a key later-line treatment option for metastatic colorectal cancer. However, the side effects, particularly proteinuria (excess protein in the urine), often force doctors to temporarily pause bevacizumab treatment. It was previously unclear how these pauses in bevacizumab treatment impacted patient survival. In this study, we analyzed the medical records of 101 patients to investigate this issue. We found that patients who received at least 70% of the originally planned amount of bevacizumab had significantly better tumor control and showed a trend toward living longer than those who received less than 70%. We also found that experiencing severe proteinuria during earlier treatments strongly predicted its return during current therapy. Our findings suggest that careful management of proteinuria throughout all stages of treatment is essential for the safe continuation of bevacizumab and the maximization of survival benefits.

## 1. Introduction

Trifluridine/tipiracil (FTD/TPI) is a later-line treatment option for metastatic colorectal cancer (mCRC) [1]. Recently, the SUNLIGHT trial, a phase III clinical trial, demonstrated that the addition of bevacizumab (Bev) to FTD/TPI monotherapy significantly prolonged the overall survival (OS) and progression-free survival (PFS) [2]. Consequently, FTD/TPI + Bev combination therapy has been established as a useful regimen for later-line treatment of mCRC. However, because angiogenesis inhibitors such as Bev, ramucirumab, and aflibercept are widely used in the frontline setting [3,4,5,6], their administration across multiple lines can lead to the accumulation of renal toxicity, frequently necessitating treatment interruption or discontinuation due to severe proteinuria. While a previous study has demonstrated that doubling the dose of Bev beyond progression can improve treatment efficacy [7], it has yet to be investigated how frequently cumulative nephrotoxicity leads to Bev interruption or discontinuation in real-world settings, and to what extent this decline in dose intensity impairs the efficacy of FTD/TPI + Bev combination therapy. Furthermore, to date, no study has provided real-world evidence demonstrating the actual clinical impact of these dose reductions. Therefore, this study aimed to evaluate the impact of Bev RDI on the therapeutic efficacy of FTD/TPI + Bev in a real-world setting.

## 2. Materials and Methods

### 2.1. Study Design and Patients

This was a single-center, retrospective cohort study that included consecutive patients who received at least one cycle of FTD/TPI + Bev combination therapy for mCRC refractory or intolerant to frontline treatments (regimens containing fluoropyrimidines, oxaliplatin, irinotecan, anti-EGFR antibodies, and angiogenesis inhibitors) at Osaka Metropolitan University Hospital between April 2016 and May 2025. Among the 104 patients who received FTD/TPI plus bevacizumab therapy, 3 patients with missing clinical data were excluded, resulting in a final cohort of 101 patients for the analysis.

### 2.2. Treatment Regimen and Definition of Relative Dose Intensity (RDI)

The treatment regimen consisted of a 28-day cycle, in which FTD/TPI 35 mg/m^2^ was administered orally twice daily on days 1–5 and 8–12, and Bev 5 mg/kg was administered via intravenous infusion every 2 weeks. The relative dose intensity (RDI) of Bev and FTD/TPI was calculated as the ratio of the actual cumulative dose to the planned standard dose over the entire period from treatment initiation to discontinuation. Based on the RDI cutoff values described later, the eligible patients were classified into two groups: a high-RDI group and a low-RDI group.

### 2.3. Interruption Criteria and Proteinuria Assessment

FTD/TPI treatment was interrupted in the event of severe hematological or non-hematological toxicity. Bev was administered according to the severity of hypertension and proteinuria. Adverse events were graded according to the Common Terminology Criteria for Adverse Events (CTCAE) version 5.0 [8].

Specifically, proteinuria was routinely monitored using a qualitative urine dipstick test before each bevacizumab administration. A quantitative evaluation using the urine protein-to-creatinine ratio (UPCR) was performed when deemed necessary by the attending physician. In this study, severe proteinuria, which typically necessitated the interruption of bevacizumab, was defined as Grade ≥ 3, corresponding to a urine dipstick reading of ≥3+ or a UPCR of ≥2.0 g/g creatinine. Bev was also interrupted concomitantly whenever FTD/TPI was interrupted.

### 2.4. Endpoints and Assessments

Clinical data were retrospectively collected from medical records. The primary endpoints were PFS, OS, and disease control rate (DCR). Tumor response was evaluated according to the Response Evaluation Criteria in Solid Tumors (RECIST) version 1.1 [9]. To ensure a rigorous evaluation of efficacy, patients who experienced clinical disease progression before the scheduled radiological evaluation were classified as having progressive disease (clinical PD). Tumor response was routinely evaluated every 8–10 weeks.

### 2.5. Statistical Analysis

Statistical analyses were performed using EZR (ver. 1.68, Saitama Medical Center, Jichi Medical University, Saitama, Japan). EZR is a graphical user interface for R (The R Foundation for Statistical Computing, Vienna, Austria) designed to add frequently used statistical functions [10]. Continuous variables were compared using the Mann–Whitney U test, and categorical variables were evaluated using the chi-square test or Fisher’s exact test, as appropriate. Survival curves were generated using the Kaplan–Meier method, and comparisons between groups were conducted using the log-rank test. To rigorously identify any independent prognostic factors and adjust for potential confounding effects, multivariate Cox proportional hazards regression analyses were performed for both PFS and OS. For PFS, the multivariate model included variables such as bevacizumab RDI (≥70% vs. <70%), Eastern Cooperative Oncology Group Performance Status (ECOG PS), prior anti-VEGF exposure, baseline renal function (eGFR), primary tumor sidedness, RAS mutation status, and the duration of the prior regimen. For OS, to prevent the number of variables from becoming excessively large relative to the limited number of events and to avoid overfitting, the number of covariates was restricted. Consequently, the OS multivariate model included only bevacizumab RDI, ECOG PS, history of prior angiogenesis inhibitor use, and the duration of the prior regimen. Furthermore, to account for any potential immortal time bias and reverse causation, whereby early progression or deterioration inevitably leads to a lower RDI, we conducted a multivariate landmark analysis of PFS and OS 60 days after treatment initiation. To identify the predictive factors associated with a reduced bevacizumab RDI (<70%) and the incidence of severe proteinuria (≥3+), logistic regression analyses were performed, and the results were expressed as odds ratios (ORs) with corresponding 95% confidence intervals (CIs).

### 2.6. Ethical Considerations

This study was conducted in compliance with the ethical principles of the Declaration of Helsinki and was approved by the Ethics Committee of Osaka City University (currently Osaka Metropolitan University) (approval number: 2020-026). Because this was a retrospective observational study utilizing only existing clinical data, the requirement to obtain direct written informed consent from patients was waived. Instead, an opt-out method was adopted, whereby information regarding the purpose and details of the study was published on the hospital’s website, guaranteeing patients the opportunity to refuse the use of their data for research purposes. Furthermore, during the data extraction and analysis, information that could directly identify individuals, such as names and medical record numbers, was removed to ensure the strict protection of patient privacy.

## 3. Results

### 3.1. Establishment of the RDI Cutoff Value for Bev

**Figure 1:**
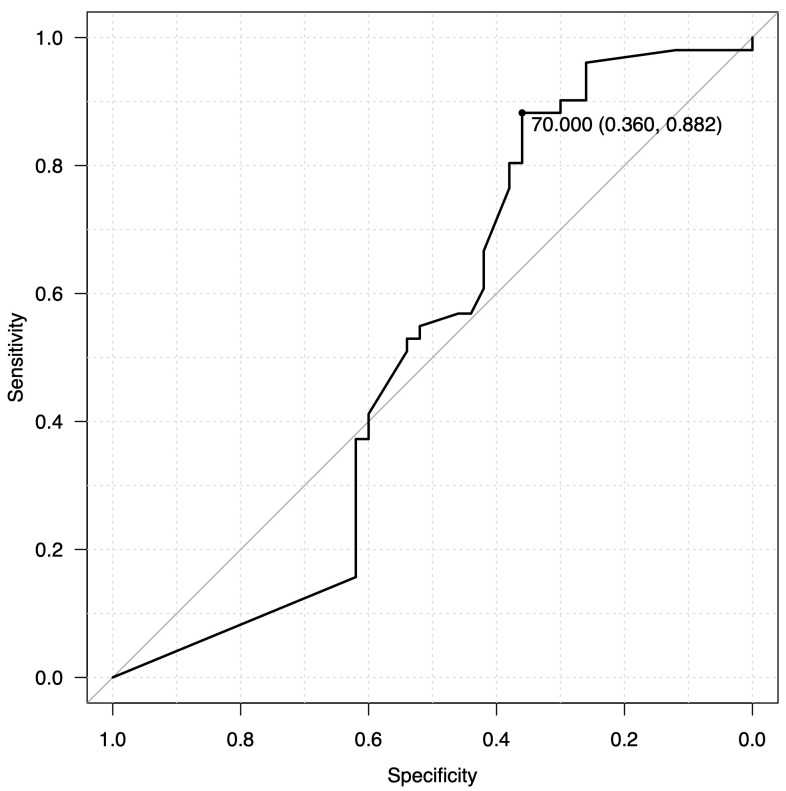
Receiver-operating-characteristic curve for determining the optimal cutoff value of the relative dose intensity of bevacizumab. In an analysis of disease progression at 108 days (the median progression-free survival for all patients) as the endpoint, the optimal cutoff value calculated using the Youden index was 70%. The area under the curve was 0.519 (95% confidence interval: 0.401–0.638; sensitivity 88.2%, specificity 36.0%). The solid gray diagonal line represents the reference line.

To evaluate the impact of Bev RDI on prognosis, the optimal cutoff value was determined using a receiver-operating-characteristic (ROC) curve. In an analysis with disease progression at 108 days (the median PFS of all patients) as the endpoint (Figure 1), the RDI value that maximized the Youden index was 70%. The area under the curve was 0.519 (95% confidence interval: 0.401–0.638); therefore, the cutoff value for this study was set at 70%.

### 3.2. Patient Characteristics

As a result of classifying the 101 eligible patients based on the Bev RDI cutoff value of 70%, 75 patients (74.3%) were assigned to the high-RDI group and 26 patients (25.7%) to the low-RDI group. The patient characteristics of both groups are comprehensively presented in Table 1. No significant differences were observed between the two groups in any of the baseline characteristics, including age, sex, ECOG PS, comorbidities, baseline proteinuria, primary tumor location, histological type, RAS mutation status, metastatic burden, number of prior regimens, details of prior anti-VEGF/angiogenesis therapy (including history, type, duration, and reason for discontinuation), and median RDI of FTD/TPI.

### 3.3. Therapeutic Efficacy

**Figure 2:**
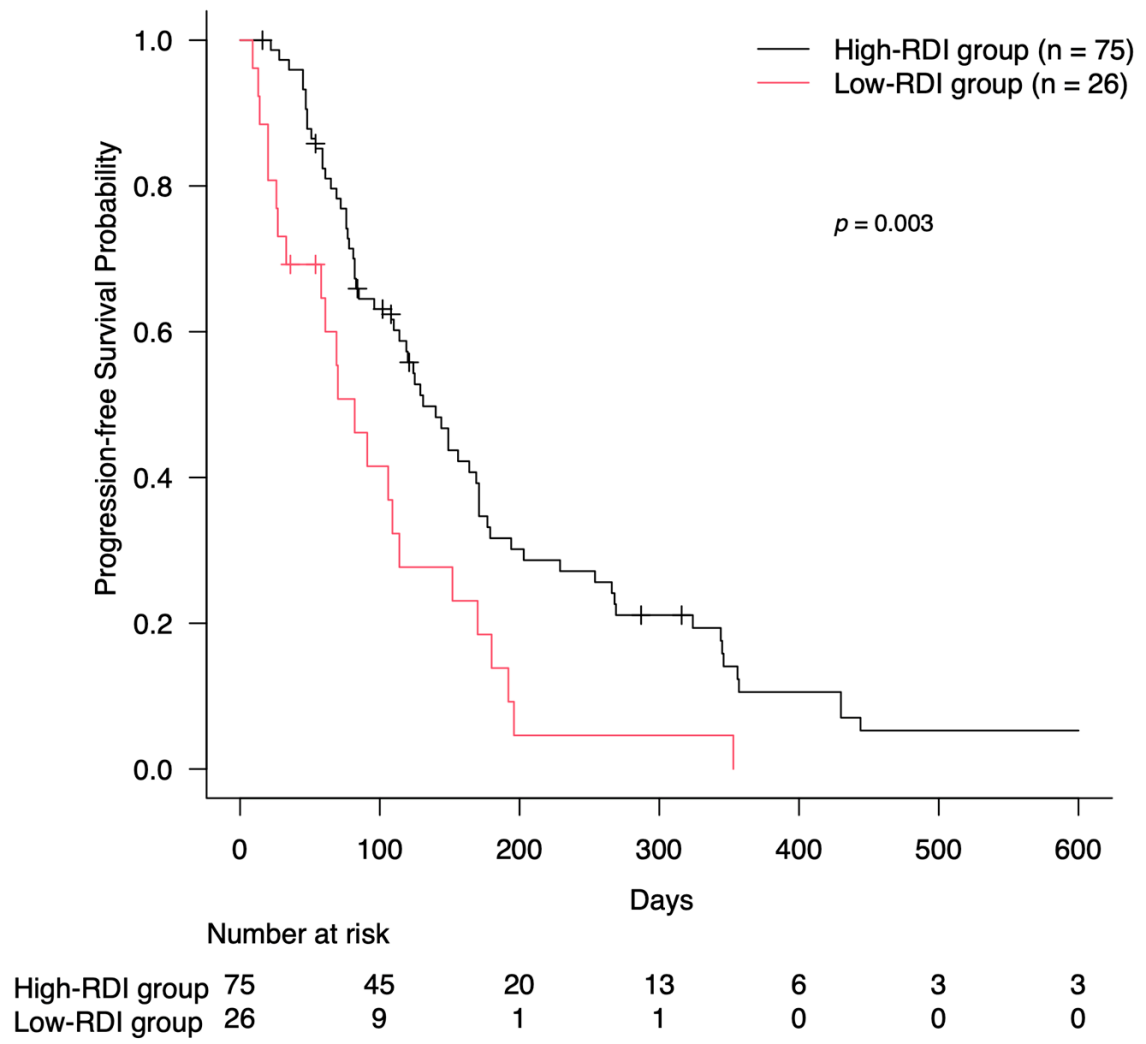
Kaplan–Meier curves for progression-free survival (PFS). Comparison of PFS between the high relative dose intensity (RDI) group (black line, n = 75) and the low-RDI group (red line, n = 26). The median PFS was 131 days in the high-RDI group and 82 days in the low-RDI group. The log-rank test was used to compare the survival curves between the groups (*p* = 0.003). The hazard ratio (HR) and 95% confidence interval (CI) were calculated using a univariate Cox proportional hazards model (HR = 2.08; 95% CI: 1.28–3.39).

**Figure 3:**
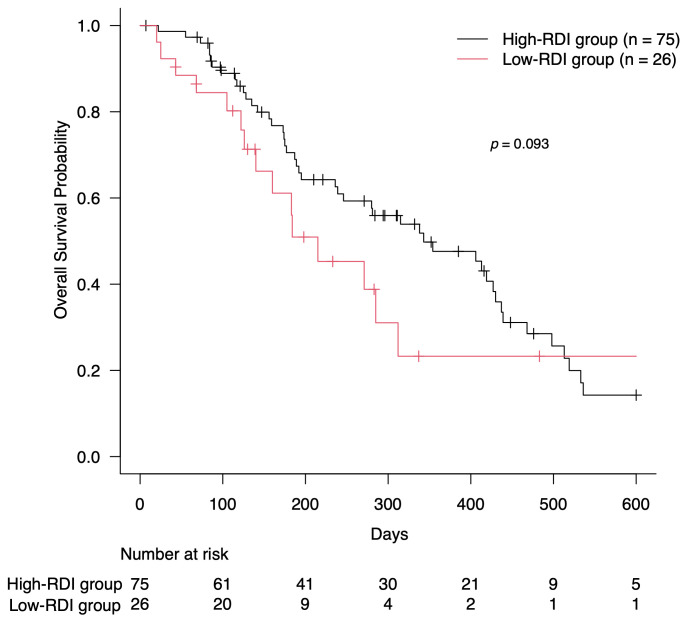
Kaplan–Meier curves for overall survival (OS). Comparison of OS between the high relative dose intensity (RDI) group (black line, n = 75) and the low-RDI group (red line, n = 26). The median OS was 343 days in the high-RDI group and 215 days in the low-RDI group. The log-rank test was used to compare the survival curves between the groups (*p* = 0.093). The hazard ratio (HR) and 95% confidence interval (CI) were calculated using a univariate Cox proportional hazards model (HR = 1.63; 95% CI: 0.92–2.91).

The median PFS was 131 days in the high-RDI group and 82 days in the low-RDI group, demonstrating a significant prolongation in the high-RDI group (*p* = 0.003; Figure 2). Similarly, the median OS was 343 days in the high-RDI group and 215 days in the low-RDI group. Although this did not reach statistical significance, a favorable trend toward improved OS was observed in the high-RDI group (*p* = 0.093; Figure 3). Furthermore, the DCR was significantly higher in the high-RDI group (46.7%) than in the low-RDI group (23.1%) (*p* = 0.040; Table 2).

### 3.4. Analysis of RDI as a Continuous Variable

The bevacizumab RDI was also analyzed as a continuous variable using a Cox proportional hazards model. The analysis demonstrated that a decrease in continuous RDI was significantly associated with a higher risk of disease progression (HR = 1.015; 95% CI: 1.003–1.027; *p* = 0.011). For OS, the hazard ratio for a decrease in continuous RDI was 1.010 (95% CI: 0.997–1.022; *p* = 0.130).

### 3.5. Multivariable Analysis for Survival

To evaluate the independent prognostic impact of the bevacizumab RDI, multivariate Cox proportional hazards models were utilized, incorporating relevant clinical confounders (Table 3). The multivariate analysis confirmed that a low bevacizumab RDI (<70%) was a strong independent risk factor for shorter PFS (HR = 1.99; 95% CI: 1.19–3.31; *p* = 0.009). A similar trend was observed for OS, although it did not reach statistical significance (HR = 1.66; 95% CI: 0.92–2.97; *p* = 0.090).

### 3.6. Landmark Analysis for Survival Outcomes

To address the potential for immortal time bias, a multivariate landmark analysis was performed at 60 days (Supplementary Table S1). Even after excluding patients with early events, a low bevacizumab RDI (<70%) remained an independent risk factor for shorter PFS (HR = 2.00; 95% CI: 1.08–3.72; *p* = 0.027). For OS, statistical significance was not reached, likely due to insufficient statistical power resulting from the limited sample size (HR = 1.49; 95% CI: 0.79–2.80; *p* = 0.219).

### 3.7. Analysis of Factors Associated with Decreased Bev RDI

When investigating the causes of dose reduction, interruption, or discontinuation among the 26 patients in the low-RDI group, the most common reason was proteinuria, which was observed in 12 patients (46.2%) (Table 4). In addition, a logistic regression analysis was conducted to identify factors contributing to a reduced bevacizumab RDI (<70%) (Table 5). The development of proteinuria of ≥3+ on urine dipstick during prior treatment showed a tendency toward an insufficient Bev dose, although it did not reach statistical significance (OR = 2.76; 95% CI: 0.89–8.62; *p* = 0.080). For the other factors examined, including estimated Glomerular Filtration Rate (eGFR) at the initiation of FTD/TPI + Bev, no significant association with an insufficient Bev dose was observed.

### 3.8. Predictive Factors for the Development of Proteinuria During FTD/TPI + Bev Therapy

Next, a logistic regression analysis was performed to identify predictive factors for the development of severe proteinuria (≥3+) during the current treatment (Table 6). The results showed that a history of proteinuria of ≥3+ on urine dipstick during prior antiangiogenic therapy was significantly correlated with the development of proteinuria of ≥3+ on urine dipstick during the current treatment (OR = 17.70; 95% CI: 4.84–65.00; *p* < 0.001). Furthermore, in patients who experienced proteinuria (≥3+ on urine dipstick) during frontline treatment, the median time (range) to the development of proteinuria during the current treatment was 28 days (14–399), indicating a tendency to develop severe proteinuria relatively early. Furthermore, male sex was also identified as a significant factor for severe proteinuria (OR = 3.23; 95% CI: 1.05–10.00; *p* = 0.040).

## 4. Discussion

In patients with mCRC receiving FTD/TPI + Bev combination therapy, maintaining a Bev RDI of ≥70% was significantly associated with prolonged PFS and a favorable trend toward improved OS. The primary cause for the reduction in RDI was treatment interruption due to proteinuria, and the development of proteinuria during this treatment was strongly associated with a history of proteinuria during prior treatments. These results suggest the importance of long-term management of renal toxicity across treatment lines.

The efficacy of this combination therapy has been established in the SUNLIGHT trial [2]. However, this trial did not report the detailed impact of the dose intensity of Bev on treatment outcomes. In this study, the most frequent direct clinical factor for a decreased Bev RDI was treatment interruption due to proteinuria. In particular, a history of severe proteinuria (≥3+ on urine dipstick) induced by angiogenesis inhibitors during prior treatment showed a notable trend toward a decreased Bev RDI during the current treatment. These findings go beyond the self-evident causal relationship that adverse events simply lead to RDI reduction, and clinically support the hypothesis that renal damage caused by angiogenesis inhibitors accumulates across treatment lines, thereby limiting the feasibility of later-line treatments.

Bev is a monoclonal antibody that specifically binds to vascular endothelial growth factor (VEGF) [11]. Because VEGF receptors are primarily expressed on endothelial cells in the renal glomerulus [12,13,14], Bev administration is considered to induce glomerular endothelial injury [15,16]. Pathologically, changes resembling renal-limited thrombotic microangiopathy (TMA), such as thrombus formation within glomerular capillaries and endothelial cell swelling, have been reported [17,18]. In addition to hypertension, which is a known side effect of Bev, proteinuria is known to occur due to this renal-limited endothelial injury. These findings have also been demonstrated by Eremina et al. [19].

Clinically, proteinuria tends to improve upon the interruption of Bev administration [20], indicating that renal injury caused by Bev is fundamentally reversible. This recovery is presumed to depend on the plasticity of the kidney and repair capacity of glomerular endothelial cells. However, cases requiring time for recovery have also been reported [21], and this repair capacity is influenced by the patient’s baseline renal function and comorbidities (such as hypertension and diabetes) [22]. The crucial concept here is the renal functional reserve [23]. Even when proteinuria appears during prior treatment and decreases over time, the improvement in proteinuria and recovery of renal function do not necessarily reflect the histological repair of the kidney. During this period, endothelial damage may not have fully resolved, and the remaining nephrons are considered to be in a state of compensatory hyperfiltration, where the glomerular filtration rate per nephron is increased. Consequently, the renal functional reserve against new stress may be diminished. Previous reports have shown an increased risk of incident chronic kidney disease (CKD) following Bev administration [24], which can be interpreted as corroborating an irreversible functional deficit after acute kidney injury and an increased future risk of developing CKD. The strong correlation between a history of prior proteinuria and the development of proteinuria in subsequent treatments demonstrated in this study can be considered clinical data that strongly supports this theory of reduced renal functional reserve and accumulation of injury. Furthermore, the finding that patients who previously exhibited severe proteinuria were prone to develop proteinuria again, despite having comparable eGFR levels at the initiation of FTD/TPI + Bev, is presumed to be due to differences in this renal functional reserve. However, as this study did not include direct pathological or specific biomarker evaluations, these microscopic mechanisms could not be confirmed definitively. Therefore, the concepts of depletion of the renal functional reserve, cumulative endothelial injury, and the potential prophylactic benefit of renin-angiotensin system or sodium–glucose cotransporter 2 (SGLT2) inhibitors are suggested as potential underlying mechanisms that warrant further investigation. Our analysis also identified male sex as a potential risk factor for developing severe proteinuria. Although the exact reasons for this male predominance remain unclear, gender differences in underlying vascular conditions, such as hypertension or arteriosclerosis, might contribute to this susceptibility [25,26]. Therefore, its true clinical significance warrants further investigation in larger cohorts.

In the current pharmacotherapy for mCRC, angiogenesis inhibitors are key drugs across all lines of treatment [27], and their continued use is essential for prognostic improvement. Hypertension can often be managed with concomitant antihypertensive drugs, thereby allowing chemotherapy to continue. In contrast, treatment interruption is the only effective measure for managing proteinuria [28]. Therefore, to maintain an effective treatment intensity in later lines of therapy, it is necessary to manage renal toxicity from a long-term perspective, starting with frontline treatment. Establishing routine urine monitoring protocols is therefore essential for the early detection of subclinical renal injury. Furthermore, given a previous report demonstrating that strict blood pressure control can reduce the risk of proteinuria during bevacizumab treatment [29], mitigating intraglomerular pressure through strict blood pressure management and the proactive administration of renin-angiotensin system inhibitors (ACE inhibitors or ARBs) might represent a crucial strategy for controlling VEGF inhibitor-induced proteinuria and maintaining the bevacizumab RDI [30]. The results of this study suggest that for high-risk patients with a history of proteinuria, these proactive interventions, alongside the potential use of SGLT2 inhibitors for renoprotection [31,32], may maximize the ultimate therapeutic efficacy. Furthermore, prospective interventional studies are warranted to evaluate whether such proactive proteinuria management can successfully maintain bevacizumab RDI and translate into improved clinical outcomes.

This study has some limitations. First, it was a single-center, retrospective study with a relatively small sample size, particularly in the low-RDI group, which may have introduced some selection bias, residual confounding, and limited generalizability. Second, the 70% cutoff value for bevacizumab RDI was derived retrospectively using an ROC analysis in this specific cohort. Given its relatively weak discriminatory performance and lack of independent validation in external cohorts, this threshold should be interpreted as an exploratory finding, and large-scale studies are warranted to validate the optimal cutoff value. Third, the calculation of RDI over the entire treatment period introduces a risk of immortal time bias and reverse causation. As this is an observational study, we cannot exclude the possibility that patients with less aggressive disease or a better performance status were able to continue treatment for a longer duration, which consequently contributed to maintaining a higher RDI. Although we attempted to minimize this impact using a landmark analysis, these biases cannot be entirely eliminated. Fourth, although our clinical data suggest an accumulation of renal endothelial cell injury, direct histological evaluations were not performed to confirm this underlying mechanism. Fifth, the evaluation of proteinuria relied on qualitative urine dipstick testing rather than on more precise quantitative measurements. Finally, the BRAF mutation status could not be included in the multivariate analysis because of extensive missing data, as it was not routinely tested during the earlier periods of this cohort.

## 5. Conclusions

In FTD/TPI + Bev combination therapy, maintaining the Bev dose (RDI ≥70%) is an important factor associated with improved therapeutic efficacy. The greatest cause of a decreased RDI was proteinuria, and the risk of its development correlated with a history of proteinuria during prior treatment. Proteinuria management based on a long-term perspective across treatment lines may lead to maximizing the therapeutic efficacy of this therapy.

## Figures and Tables

**Table 1:** Patient Characteristics.

	All (n = 101)	High-RDI Group (n = 75)	Low-RDI Group (n = 26)	*p*-Value
Age (years)				0.616
median (range)	69 (36–88)	68 (36–86)	70 (47–88)	
Sex, n (%)				0.654
Male	51 (50.5)	39 (52.0)	12 (46.2)	
Female	50 (49.5)	36 (48.0)	14 (53.8)	
ECOG PS, n (%)				0.276
0, 1	91 (90.1)	69 (92.0)	22 (84.6)	
≥2	10 (9.9)	6 (8.0)	4 (15.4)	
Hypertension, n (%)				0.250
Yes	43 (42.6)	29 (38.7)	14 (53.8)	
No	58 (57.4)	46 (61.3)	12 (46.2)	
Diabetes mellitus, n (%)				0.445
Yes	26 (25.7)	21 (28.0)	5 (19.2)	
No	75 (74.3)	54 (72.0)	21 (80.8)	
Chronic kidney disease, n (%) Yes (eGFR <60 mL/min/1.73 m^2^)	29 (28.7)	20 (26.7)	9 (34.6)	0.459
No (eGFR ≥60 mL/min/1.73 m^2^)	72 (71.3)	55 (73.3)	17 (65.4)	
Baseline proteinuria, n (%)				0.136
0, ± or 1+	89 (88.1)	69 (92.0)	20 (76.9)	
≥2+	11 (10.9)	6 (8.0)	5 (19.2)	
Unknown	1 (1.0)	0 (0.0)	1 (3.9)	
Primary tumor location, n (%)				0.815
Right	30 (29.7)	23 (30.7)	7 (26.9)	
Left	71 (70.3)	52 (69.3)	19 (73.1)	
Histological type, n (%)				0.190
Tub1/tub2	92 (91.1)	68 (90.7)	24 (92.3)	
Por/sig/muc	7 (6.9)	7 (9.3)	0 (0.0)	
Unknown	2 (2.0)	0 (0.0)	2 (7.7)	
RAS status, n (%)				0.647
Wild type	45 (44.6)	35 (46.7)	10 (38.5)	
Mutant type	53 (52.5)	39 (52.0)	14 (53.8)	
Unknown	3 (2.9)	1 (1.3)	2 (7.7)	
Metastatic burden, n (%)				0.816
1 organ	38 (37.6)	29 (38.7)	9 (34.6)	
≥2 organs	63 (62.4)	46 (61.3)	17 (65.4)	
Number of prior regimens, n (%)				0.173
3	64 (63.4)	45 (60.0)	19 (73.1)	
4	21 (20.8)	19 (25.3)	2 (7.7)	
≥5	16 (15.8)	11 (14.7)	5 (19.2)	
History of prior angiogenesis inhibitor use, n (%)				0.200
Yes	75 (74.3)	53 (70.7)	22 (84.6)	
No	26 (25.7)	22 (29.3)	4 (15.4)	
Type of prior anti-VEGF/angiogenesis agent, n (%)				0.190
Bevacizumab	31 (30.7)	25 (33.3)	6 (23.1)	
Ramucirumab	30 (29.7)	18 (24.0)	12 (46.2)	
Aflibercept	1 (1.0)	1 (1.3)	0 (0.0)	
Regorafenib	13 (12.9)	9 (12.0)	4 (15.4)	
None	26 (25.7)	22 (29.3)	4 (15.4)	
Duration of immediate prior anti-VEGF therapy (days)				0.913
median (range)	171 (13–711)	140 (13–650)	178 (13–711)	
Reason for discontinuation of prior therapy, n (%)				0.088
Progressive disease (PD)	89 (88.1)	67 (89.3)	22 (84.6)	
Adverse events	9 (8.9)	6 (8.0)	3 (11.5)	
others	3 (3.0)	2 (2.7)	1 (3.8)	
FTD/TPI RDI (%)				0.290
median (range)	82.5 (19–100)	85.0 (19–100)	76.0 (40–100)	

ECOG PS, Eastern Cooperative Oncology Group performance status; eGFR, estimated glomerular filtration rate; tub, tubular adenocarcinoma; por, poorly differentiated adenocarcinoma; sig, signet-ring cell carcinoma; muc, mucinous adenocarcinoma; RAS, rat sarcoma viral oncogene homolog; VEGF, vascular endothelial growth factor; FTD/TPI, trifluridine/tipiracil; RDI, relative dose intensity.

**Table 2:** Comparison of disease control rate.

	High-RDI Group (n = 75)	Low-RDI Group (n = 26)	*p*-Value
Complete Response (CR), n (%)	0 (0.0%)	0 (0.0%)	
Partial Response (PR), n (%)	6 (8.0%)	0 (0.0%)	
Stable Disease (SD), n (%)	29 (38.7%)	6 (23.1%)	
Progressive Disease (PD), n (%)	40 (53.3%)	20 (76.9%)	
Disease Control Rate (CR + PR + SD), n (%)	35 (46.7%)	6 (23.1%)	0.040

CR, complete response; PR, partial response; SD, stable disease; PD, progressive disease; RDI, relative dose intensity. Patients whose best overall response was not evaluable (NE) were classified as having PD (n = 1 in each group).

**Table 3:** Univariate and multivariate analyses of factors associated with progression-free survival and overall survival.

Factors				
	Univariate		Multivariate	
	HR (95% CI)	*p* Value	HR (95% CI)	*p* Value
Progression-free survival				
Bev RDI (≥70% vs. <70%)				
High (≥70%)	1.00 (Reference)		1.00 (Reference)	
Low (<70%)	2.08 (1.28–3.39)	0.003	1.99 (1.19–3.31)	0.009
ECOG PS (≥2 vs. 0, 1)	2.56 (1.27–5.13)	0.008	2.64 (1.26–5.52)	0.010
History of prior angiogenesis inhibitor use (Yes vs. No)	0.89 (0.54–1.47)	0.655	1.00 (0.59–1.71)	0.994
Duration of prior regimen (days)	1.00 (1.00–1.00)	0.454	1.00 (1.00–1.00)	0.379
Decreased eGFR at the initiation of FTD/TPI + Bev (<60 vs. ≥60)	1.10 (0.69–1.76)	0.691	0.98 (0.60–1.61)	0.932
Primary tumor location (Left vs. Right)	1.06 (0.67–1.67)	0.814	0.98 (0.60–1.61)	0.932
RAS status (Wild vs. Mutant)	1.06 (0.69–1.62)	0.804	1.10 (0.67–1.79)	0.715
Overall survival				
Bev RDI (≥70% vs. <70%)				
High (≥70%)	1.00 (Reference)		1.00 (Reference)	
Low (<70%)	1.63 (0.92–2.91)	0.096	1.66 (0.92–2.97)	0.090
ECOG PS (≥2 vs. 0, 1)	1.99 (0.85–4.66)	0.115	1.82 (0.77–4.32)	0.172
History of prior angiogenesis inhibitor use (Yes vs. No)	1.37 (0.76–2.45)	0.292	1.41 (0.78–2.54)	0.257
Duration of prior regimen (days)	1.00 (1.00–1.00)	0.413	1.00 (1.00–1.00)	0.320
Decreased eGFR at the initiation of FTD/TPI + Bev (<60 vs. ≥60)	0.90 (0.51–1.61)	0.730		
Primary tumor location (Left vs. Right)	1.19 (0.69–2.05)	0.540		
RAS status (Wild vs. Mutant)	1.43 (0.86–2.37)	0.165		

HR, hazard ratio; CI, confidence interval; Bev, bevacizumab; RDI, relative dose intensity; ECOG PS, Eastern Cooperative Oncology Group Performance Status; eGFR, estimated glomerular filtration rate. Blank cells in the multivariate analysis for overall survival indicate variables that were excluded from the model to prevent the number of variables from becoming excessively large relative to the number of events, thereby avoiding overfitting.

**Table 4:** Reasons for bevacizumab interruption.

Reasons for Bevacizumab Interruption (low-RDI Group)	n (%)
Proteinuria	12 (46.2)
General fatigue	5 (19.2)
Hematotoxicity	4 (15.4)
Patient requests	3 (11.5)
Fever	2 (7.7)

RDI, relative dose intensity.

**Table 5:** Univariate analysis of factors associated with a bevacizumab relative dose intensity of <70%.

Background Factors	OR	95% CI	*p* Value
History of proteinuria of ≥3+ on urine dipstick during prior anti-VEGF therapy (Yes vs. No)	2.76	0.89–8.62	0.080
Use of anti-VEGF drugs in prior treatment (Yes vs. No)	2.28	0.71–7.40	0.169
ECOG PS (≥2 vs. 0, 1)	2.09	0.54–8.09	0.285
Age (Continuous)	1.01	0.97–1.06	0.529
Sex (Female vs. Male)	1.27	0.52–3.13	0.608
RAS status (Wild type vs. Mutant type)	1.26	0.50–3.19	0.631
Primary lesion (Left vs. Right)	1.20	0.44–3.25	0.719
Decreased eGFR at the initiation of FTD/TPI + Bev (<60 mL/min/1.73 m^2^ vs. ≥60 mL/min/1.73 m^2^)	1.46	0.56–3.79	0.441

OR, odds ratio; CI, confidence interval; VEGF, vascular endothelial growth factor; ECOG PS, Eastern Cooperative Oncology Group Performance Status; FTD/TPI, trifluridine/tipiracil; eGFR, estimated glomerular filtration rate; Bev, bevacizumab.

**Table 6:** Univariate analysis of factors associated with the risk of developing proteinuria (urine dipstick ≥3+) during this treatment.

Background Factors	OR	95% CI	*p* Value
History of proteinuria of ≥3+ on urine dipstick during prior anti-VEGF therapy (Yes vs. No)	17.70	4.84–65.00	<0.001
Use of anti-VEGF drugs in prior treatment (Yes vs. No)	0.88	0.28–2.76	0.828
ECOG PS (≥2 vs. 0, 1)	0.48	0.06–4.08	0.504
Age (Continuous)	1.04	0.99–1.09	0.156
Sex (Male vs. Female)	3.23	1.05–10.00	0.040
RAS status (Wild type vs. Mutant type)	0.82	0.29–2.28	0.701
Primary lesion (Left vs. Right)	0.81	0.27–2.42	0.710
Decreased eGFR at the initiation of FTD/TPI + Bev (<60 mL/min/1.73 m^2^ vs. ≥60 mL/min/1.73 m^2^)	1.30	0.44–3.89	0.633

OR, odds ratio; CI, confidence interval; VEGF, vascular endothelial growth factor; ECOG PS, Eastern Cooperative Oncology Group Performance Status; FTD/TPI, trifluridine/tipiracil; eGFR, estimated glomerular filtration rate; Bev, bevacizumab.

## Data Availability

The data presented in this study are available on request from the corresponding author. The data are not publicly available due to privacy and ethical restrictions.

## Supplementary Materials

The following supporting information can be downloaded at: https://www.mdpi.com/article/10.3390/cancers18152429/s1, Table S1. Multivariate landmark analyses for progression-free survival and overall survival based on bevacizumab relative dose intensity (<70% vs. ≥70%) at 60 days.

## Abbreviations

The following abbreviations are used in this manuscript:

Bev	Bevacizumab
BRAF	v-Raf murine sarcoma viral oncogene homolog B
CI	Confidence interval
CKD	Chronic kidney disease
CTCAE	Common Terminology Criteria for Adverse Events
DCR	Disease control rate
ECOG PS	Eastern Cooperative Oncology Group Performance Status
eGFR	Estimated glomerular filtration rate
FTD/TPI	Trifluridine/tipiracil
HR	Hazard ratio
mCRC	Metastatic colorectal cancer
OR	Odds ratio
OS	Overall survival
PFS	Progression-free survival
RAS	Rat sarcoma viral oncogene homolog
RDI	Relative dose intensity
RECIST	Response Evaluation Criteria in Solid Tumors
ROC	Receiver operating characteristic
SGLT2	Sodium–glucose co-transporter 2
TMA	Thrombotic microangiopathy
UPCR	Urine protein-to-creatinine ratio
VEGF	Vascular endothelial growth factor
