# Supplementary material for: Impact of Bevacizumab Relative Dose Intensity and Treatment-Induced Proteinuria on the Efficacy of Trifluridine/Tipiracil Plus Bevacizumab in Metastatic Colorectal Cancer
Source: Cancers (Basel). 2026 Jul 28;18(15):2429. doi: 10.3390/cancers18152429 (PMC13465344; doi:10.3390/cancers18152429)
Supplement: Supplementary file 1 [file cancers-18-02429-s001.zip › cancers-4417223-supplementary.pdf]

Table S1. Multivariate landmark analyses for progression-free survival and overall survival based on bevacizumab relative dose intensity (<70% vs. ≥70%) at 60 days.

Landmark point	Outcomes	HR (95% CI) *	*p* value
Day 60	Progression-free survival	2.00 (1.08–3.72)	0.027
	Overall survival	1.49 (0.79–2.80)	0.219

HR, hazard ratio; CI, confidence interval.

* Hazard ratios are for the low relative dose intensity (<70%) group compared with the high relative dose intensity (≥70%) group. The multivariate models were adjusted for the covariates used in the primary analyses (Table 3).

 https://doi.org/10.3390/cancers18152429
